# A case of triple digestive tract reconstruction in chronic pancreatitis complicated with bile ductal stenosis, duodenal stenosis, and portal vein stenosis: a case report

**DOI:** 10.1186/s40792-020-00872-3

**Published:** 2020-05-26

**Authors:** Yuka Abe, Takafumi Kumamoto, Gakuryu Nakayama, Kentaro Miyake, Yasuhiro Yabushita, Yu Sawada, Yuki Homma, Kazuhisa Takeda, Ryusei Matsuyama, Itaru Endo

**Affiliations:** Department of Gastroenterological Surgery, Graduate School of Medicine, Yokohama City University, 3-9 Fukuura, Kanazawa-ku, Yokohama, 236-0004 Japan

**Keywords:** Chronic pancreatitis, Surgery, Drainage procedure, Pancreaticojejunostomy, Pancreaticoduodenectomy, Portal vein stenosis, Portal vein hypertension, Venous collateral, Biliary stricture, Common bile duct, Duodenal stenosis

## Abstract

**Background:**

Although endoscopic interventions for chronic pancreatitis are highly developed, surgery for severe complicated cases such as the coexistence of bile duct, duodenum, and portal vein stenosis is a challenging issue for surgeons. In such instances, pancreaticoduodenectomy could lead to massive intraoperative bleeding due to severe collateral veins. A surgical drainage procedure, instead of pancreatic resection, may be a reasonable and safer option in such cases, but the literature on a surgical drainage technique to resolve all obstructions of the pancreatic duct, bile duct, and duodenum at once is limited. We devised a new surgical drainage method for such cases with consideration for a possible future second surgery for newly developed pancreatic cancer because chronic pancreatitis is a well-known high-risk factor for pancreatic cancer in the long term. Here, we report this surgical procedure.

**Case presentation:**

A 55-year-old man was diagnosed with alcoholic chronic pancreatitis 15 years ago. Before surgery, he underwent regular endoscopic pancreatic stenting for pancreatic ductal stenosis for 3 years. Three months before surgery, his duodenal stenosis worsened, and he was referred to our department for surgery. Preoperative imaging revealed pancreatic and bile duct stenosis, duodenal stenosis, and portal vein stenosis. To avoid intraoperative bleeding caused by the development of collateral veins, we performed a triple drainage procedure: longitudinal pancreaticojejunostomy with coring-out of the pancreatic head, hepaticojejunostomy, and gastrojejunostomy. The patient did not develop postoperative complications, and he was discharged from the hospital on postoperative day 14. For 5 years after surgery, no abdominal pain or recurrent pancreatitis was observed.

**Conclusion:**

Our triple drainage procedure seems effective and minimally invasive for patients complicated with bile duct stenosis, duodenal stenosis, and portal vein stenosis.

## Background

The surgical treatments for chronic pancreatitis are mainly composed of two approaches: drainage procedures and pancreatic resection. Drainage procedures, such as longitudinal pancreaticojejunostomy, hepaticojejunostomy, and gastrojejunostomy, can be chosen or combined depending on the obstructive site. Pancreatic resection, such as pancreatoduodenectomy, distal pancreatectomy, and Beger’s procedure [1], can be performed depending on the type or site of the lesion. If the case is complicated simultaneously with three obstructive lesions of the pancreatic duct, bile duct, and duodenum, pancreaticoduodenectomy could be chosen [2, 3]. If the inflammation from chronic pancreatitis extends to the portal vein and the patient is also complicated with portal vein stenosis in addition to three obstructive lesions, pancreaticoduodenectomy may lead to extensive bleeding during surgery due to the development of collateral veins [4, 5]. It is also assumed that persistent clamping of the portal veins for hemostasis may lead to intestinal congestion and ischemia-reperfusion injury of the liver; thus, this method seems to be too invasive for benign diseases such as chronic pancreatitis. However, the literature on how to perform surgical drainage procedures for patients complicated with pancreatic and biliary stenosis, duodenal stenosis, and portal vein stenosis is limited. In addition, chronic pancreatitis is a risk factor for pancreatic cancer in the long-term follow-up period [6, 7], so surgery for such patients would be better to be devised considering the possibility of a second operation for pancreatic cancer.

## Case presentation

A 55-year-old male was diagnosed with alcoholic chronic pancreatitis 15 years ago. He had been admitted to the hospital three times because of pancreatic pseudocysts, and 4 years ago, he was referred to our hospital due to repeated abdominal pain. Since then, he has undergone endoscopic pancreatic stenting for pancreatic ductal stenosis every 2–3 months. Furthermore, he often needed admission because of acute pancreatitis or retrograde pancreatic infection. Three months before surgery, his condition aggravated during oral intake due to duodenal stenosis, which was identified by gastrointestinal endoscopy. Therefore, he was referred to our department for surgery.

**Fig. 1:**
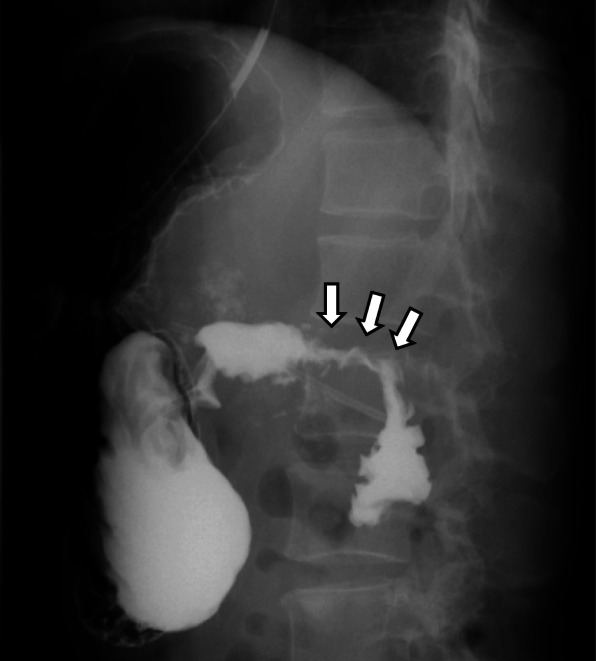
Findings of upper gastrointestinal series. Standing, 2nd oblique position. The stenosis of the superior duodenal angulus of 4 cm length was revealed (white arrow)

**Fig. 2:**
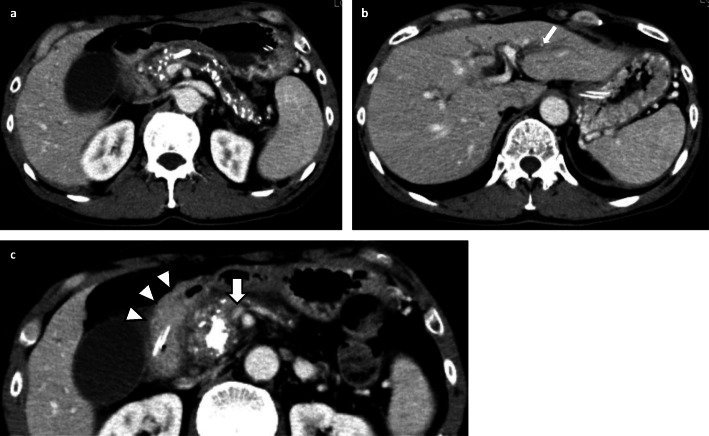
Findings of enhanced CT. Diffuse pancreatic stones and dilation of the pancreatic duct were observed (**a**). CT also showed slight dilation of the intrahepatic bile ducts (**b**), thickening of the duodenum wall (**c**, white arrow head), and stenosis of the superior mesenteric vein (**c**, white thick arrow)

**Fig. 3:**
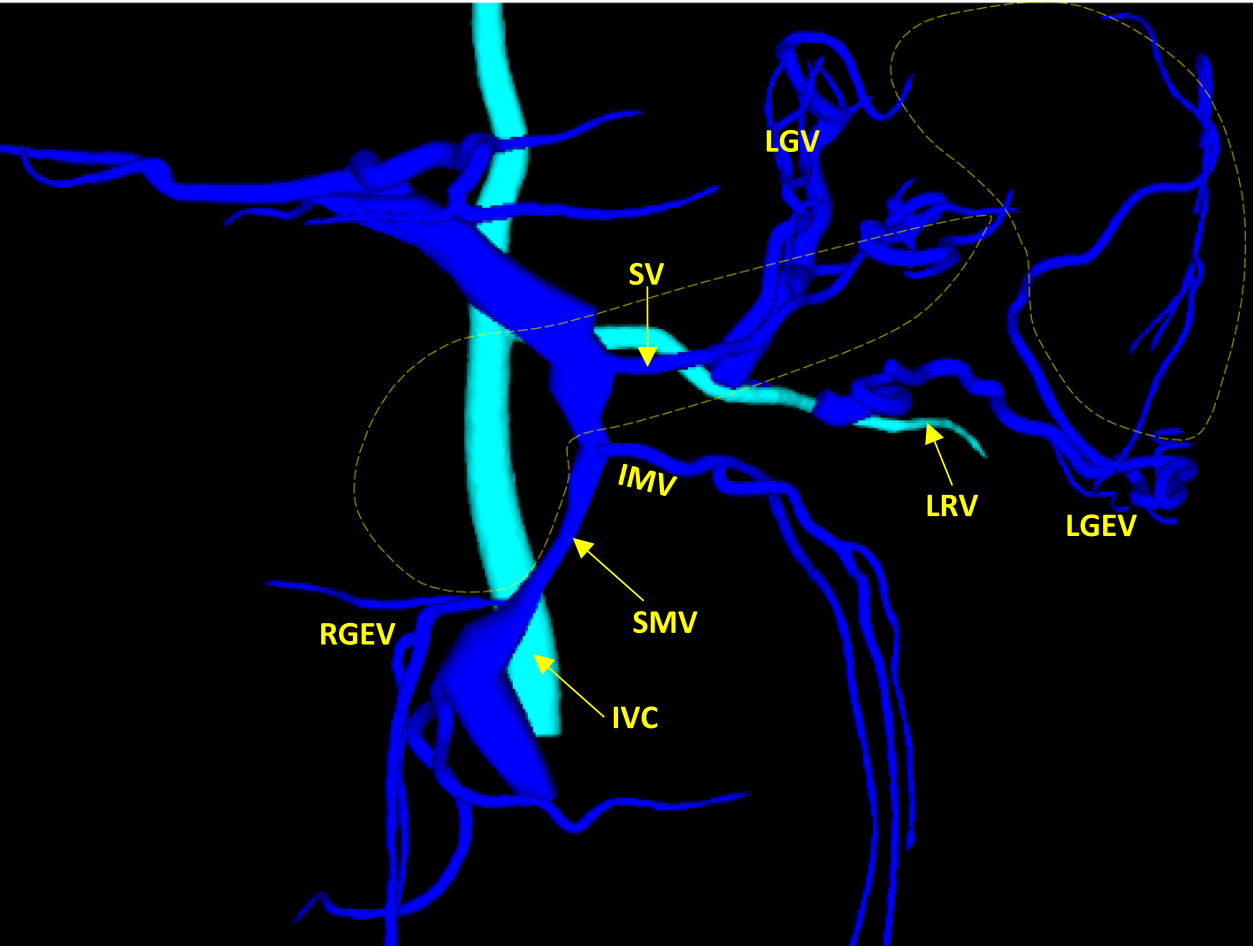
3D-CT. Development of collateral veins was observed due to stenosis of the superior mesenteric vein and the splenic vein. Collateral veins of the left gastroepiploic veins and left gastric veins were partially connected to the right renal vein

The laboratory findings showed elevated liver enzyme levels, which suggested bile duct stenosis (AST 122 U/L, ALT 221 U/L, ALP 1408 U/L, γGTP 382 U/L, T-Bill 0.4 mg/dL). The upper gastrointestinal series showed stenosis of the superior duodenal angulus (Fig. 1). Computed tomography (CT) showed diffuse pancreatic stones and dilation of the pancreatic duct at the pancreatic body and tail (Fig. 2a). CT also showed slight dilation of the intrahepatic bile ducts (Fig. 2b), thickening of the duodenum wall, and stenosis of the superior mesenteric vein (SMV) (Figs. 2c and 3), which suggested that the pancreatic inflammation spread to the bile duct, duodenum, and SMV. 3D-CT revealed that stenosis of the SMV and splenic vein caused the development of collateral veins around the left gastroepiploic veins and left gastric veins (Fig. 3). Each time endoscopic pancreatic stenting was performed, pancreatic fluid was collected, and cytology showed no malignancy. Biopsy of the duodenal mucosa also showed no malignancy. The patient was diagnosed with chronic pancreatitis refractory to endoscopic management complicated by pancreatic and biliary stenosis, duodenal stenosis, and portal vein stenosis. Initially, we planned pancreaticoduodenectomy as a definitive treatment. However, we decided to perform surgical drainage procedures at multiple sites since the development of severe collateral veins might cause massive intraoperative bleeding.

**Fig. 4:**
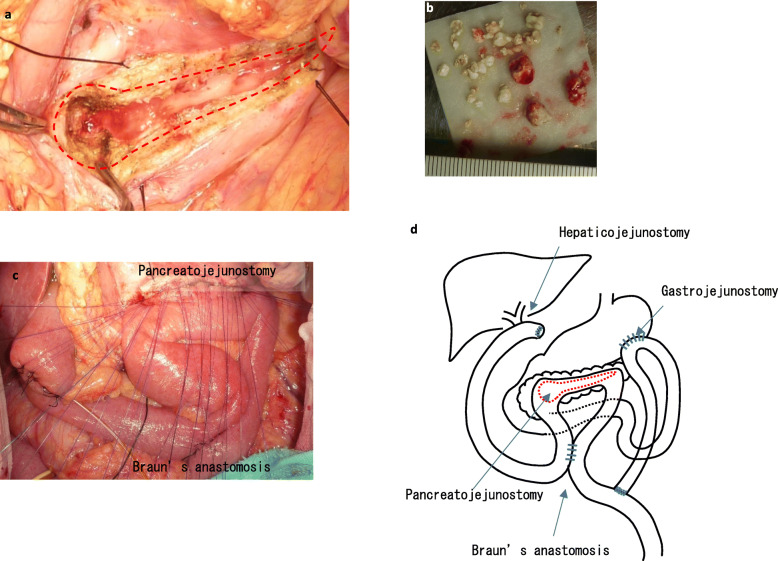
Operative findings. We made a longitudinal incision along the main pancreatic duct and excised the pancreatic head (**a**). Many pancreatic stones were removed from the pancreatic duct (**b**). After hepaticojejunostomy, we performed pancreatojejunostomy with a side-to-side anastomosis (**c**). After pancreatojejunostomy, Braun’s anastomosis was placed, and gastrojejunostomy was performed (**d**)

Intraoperative ultrasonography showed no mass lesions in the pancreas. First, we made a longitudinal incision along the main pancreatic duct and excised the inflamed parenchyma of the pancreatic head (Fig. 4a). Pancreatic stones and purulent pancreatic fluid were removed from the widely opened pancreatic duct (Fig. 4b). Then, cholecystectomy was performed, and the common bile duct was resected. The distal stump of the disconnected jejunum was lifted to facilitate hepaticojejunostomy using the ante-colic route, and longitudinal pancreatojejunostomy was performed with the distal side of the hepaticojejunostomy (Fig. 4c, d). Braun’s anastomosis was added between the hepaticojejunostomy and pancreatojejunostomy sites. Next, the distal area of the Treitz ligament was lifted for gastrojejunostomy using the ante-colic route, and enteroenterostomy of the Roux-en-Y limb was performed in the area distal to Braun’s anastomosis (Fig. 4d). The operation time was 566 min, and the amount of blood loss was 821 mL.

**Fig. 5:**
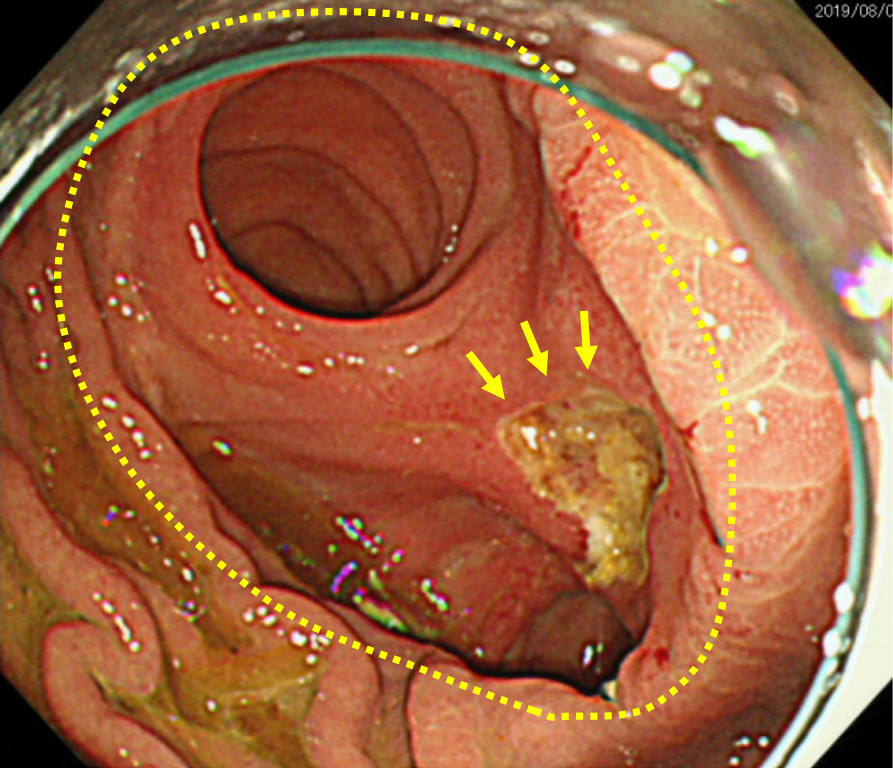
Findings of endoscopy 3 years after surgery. Anastomotic ulcer (yellow arrow) at the gastrojejunostomy (yellow circle) was found

The amylase level of the drainage fluid from the abdominal drains was 1043 U/L on postoperative day 1 and decreased day by day; the drainage tube was removed on postoperative day 4. No postoperative pancreatic fistula or intra-abdominal abscess was observed. No intestinal obstructions or any other postoperative complications occurred. The patient was discharged on postoperative day 14. One year after surgery, he was admitted to the hospital because of mild acute cholangitis. Despite the administration of an H2 blocker after surgery, he suffered from bleeding from an anastomotic ulcer at the gastrojejunostomy (Fig. 5) 3 years later. The bleeding could be stopped by the administration of proton-pump inhibitors (PPI), but because of the drug-induced liver dysfunction, we had to keep away from administration of PPI, and the bleeding from the anastomotic ulcer recurred twice.

**Fig. 6:**
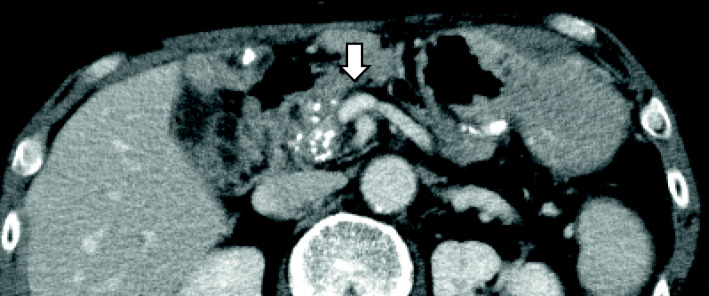
Findings of enhanced CT 3 years after surgery. The stenosis of the portal vein was improved

Five years after surgery, the patient did not experience abdominal pain at all, and acute pancreatitis did not recur. Although he has lost 5 kg in weight, the albumin level is 3.6 g/dL and HbA1c is 6.6%, which suggests that his nutrition status and diabetes status are maintained. Being performed every year, CT showed the improvement of portal vein stenosis (Fig. 6), and pancreatic cancer has not yet been detected.

## Discussion

In chronic pancreatitis (CP), stenosis of the pancreatic duct or pancreatic calculi causes pancreatic outflow disturbances, resulting in repeated abdominal pain or functional damage to the pancreas. The main purpose of CP treatment is decompression of the pancreatic duct, and the treatment of CP includes endoscopic and surgical drainage. Because CP is a progressive disease, minimally invasive therapies are recommended as an initial therapy [8]. Although endoscopic pancreatic stenting (EPS) is as effective as surgery for abdominal pain relief, EPS requires repeated hospitalizations, and it is often difficult to remove the stent in severe stenosis cases [9]. For such cases, surgical treatment should be considered. The therapeutic concept of surgery for stenosis of the pancreatic duct in CP is divided into two categories: drainage procedures and pancreatic resection. An example of a drainage procedure is longitudinal pancreaticojejunostomy. Pancreatic resections include pancreatoduodenectomy, Beger’s procedure [1], and distal pancreatectomy. Frey’s procedure [10] is a hybrid technique excising the inflammatory parenchyma of the pancreatic head and draining the pancreatic duct.

In chronic pancreatitis, inflammation of the pancreatic parenchyma often also causes stenosis of the distal common bile duct or duodenum. The incidence of biliary strictures among patients hospitalized for chronic pancreatitis ranges from 3 to 23% [11]. The surgical procedures to drain the common bile duct include choledochojejunostomy and choledochoduodenostomy [11]. Duodenal stenosis due to CP rarely occurs, the incidence of which is reportedly 1.2% [11]. The operative choices for duodenal stenosis include gastrojejunostomy and pancreaticoduodenectomy [11].

In further advanced CP cases, inflammation can spread to the portal venous system. Compression of the veins by enlarged and fibrotic pancreatic tissue causes vascular stenosis or occlusion [5]. Splenoportal venous occlusion has been reported with an incidence of 13.2% among CP patients [5]. Stenosis and occlusion of the splenoportal venous system potentially have two problems: one is the intestinal bleeding from the varices due to portal hypertension (PH), and another is the massive intraoperative bleeding because of the developed collateral veins.

Intestinal bleeding from varices due to PH is often treated successfully by interventional recanalization of obstructive veins [12, 13]. However, there are no definite criteria about the timing of inserting a portal stent. It is reported that a decrease of hepatic venous pressure gradient (HVPG) under the threshold of 12 mmHg is protective against portal hypertension-related events [14]. Therefore, it would be better to insert a portal vein stent depending on HVPG measured preoperatively or intraoperatively. However, in most of the other reports, vascular stents were placed for both benign and malignant stenosis of the portal vein when clinical symptoms are observed, such as bleeding from varices, ascites, and thrombocytopenia due to splenomegaly [15–17]. In other words, the indications for portal vein stent insertion have not been determined yet. Fortunately, our case has not suffered from bleeding of the intestinal varices, and therefore, we have not yet inserted a portal stent. If our case had complicated with variceal bleeding at the time of surgery, insertion of a portal stent should have been considered.

The development of collateral veins due to PH may lead to intraoperative bleeding. Adam et al. reported that intraoperative bleeding problems occurred in 69% of CP patients with PH, and the duration of surgery and volume of intraoperative transfusions were significantly higher in CP patients with PH than in patients without PH [5]. In such patients with PH, pancreaticoduodenectomy or Beger’s procedure often cannot be completed, and the surgical strategy needs to be changed to Frey’s procedure [4, 18].

**Fig. 7:**
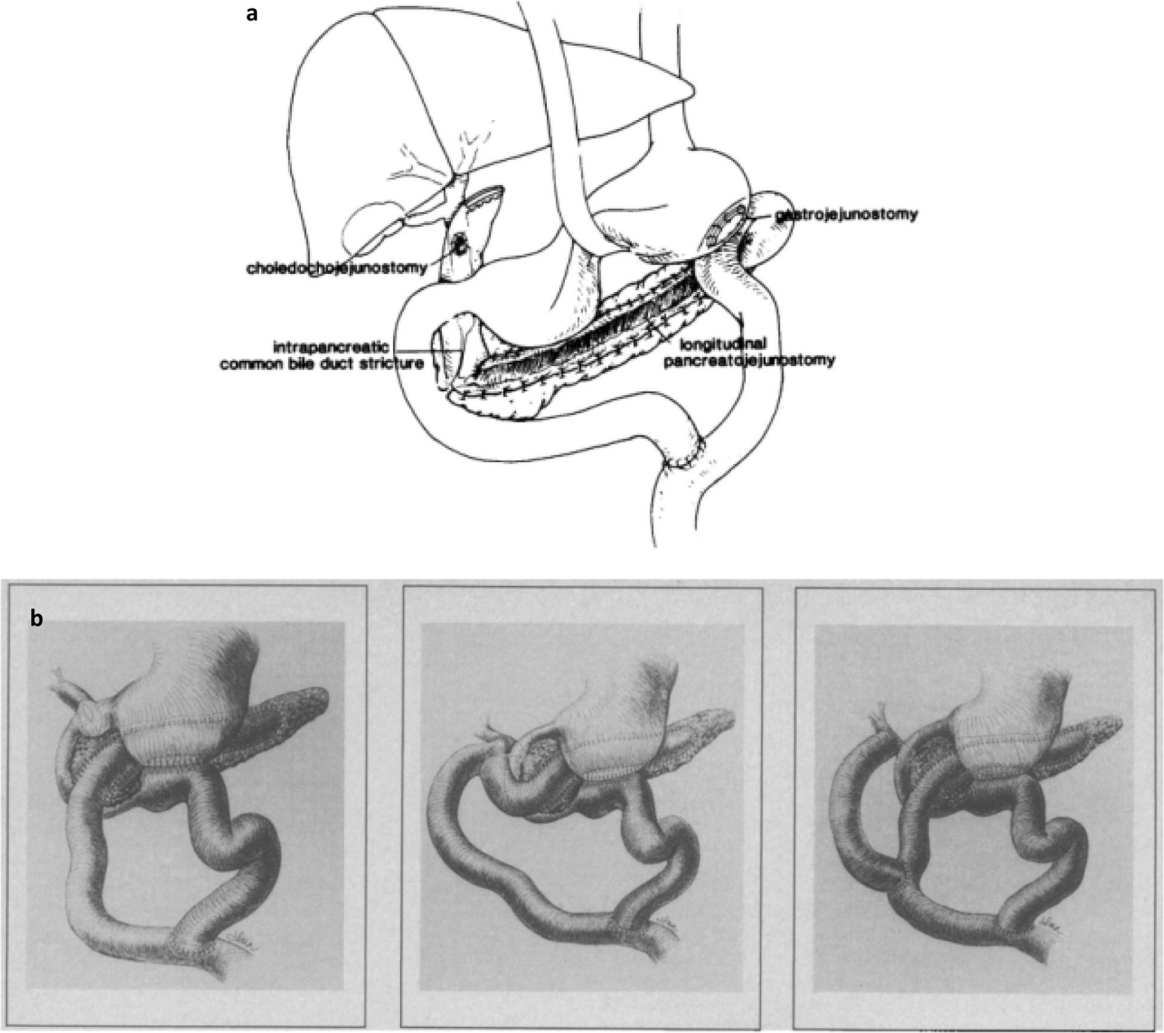
Harvey et al. [12] reported a surgical procedure with combined biliary, pancreatic, and gastric drainage (**a**). Richard et al. [13] reported three methods of treating combined obstructive complications (**b**). Permission to reproduce both figures was obtained from the Copyright Clearance Center

Our CP patient was simultaneously complicated with portal vein stenosis in addition to triple obstruction of the pancreatic duct, bile duct, and duodenum. The literature on the surgical management for such cases is extremely limited. Using the PubMed database, we identified only two studies from 1976 to 2019 with the keywords CP, drainage, duodenal obstruction, and common bile duct disease. Harvey et al. (Fig. 7a) and Richard et al. (Fig. 7b) reported a triple drainage procedure for chronic pancreatitis [18, 19]. Compared with their procedure, our method interposes Braun’s anastomosis between the hepaticojejunostomy and pancreaticojejunostomy sites and creates hepaticojejunostomy with an end-to-side anastomosis (Fig. 4d). Besides, we performed gastrojejunostomy with the jejunum distal to the Treitz ligament, which avoids bile juice from flowing directly into the stomach and may help prevent gastric cancer that results from long-term exposure to bile juice.

**Fig. 8:**
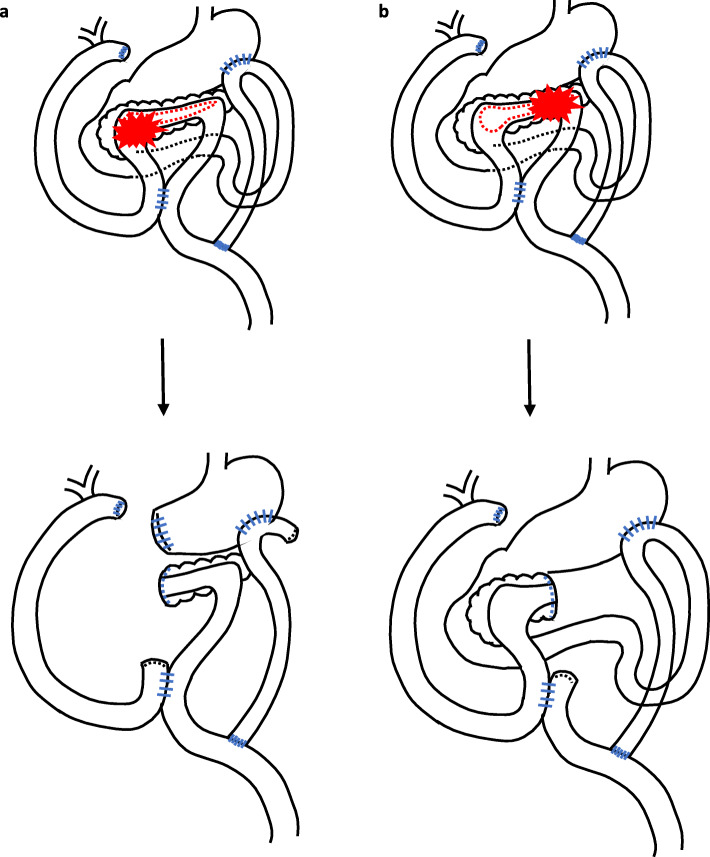
Second operation for pancreatic cancer after our procedure. If pancreaticoduodenectomy (**a**) or distal pancreatectomy (**b**) will be performed, reconstruction of the bile duct or jejunum will not be necessary

In addition, we devised this procedure to mainly consider the possibility that the patient will undergo a second surgery if he is diagnosed with metachronous pancreatic cancer in the future. Ueda et al. reported that the standardized incidence ratio of pancreatic cancer among CP patients was 11.8, and the incidence of pancreatic cancer was significantly lower in patients who had received surgery for CP than in those who had not undergone surgery [6]. Zheng et al. also mentioned that the surgical intervention plays a protective role in the development of pancreatic cancer from CP, but they reported that the time interval to surgery for CP is an independent risk factor for pancreatic cancer development after surgery [20]. Therefore, we should still keep in mind the risk of pancreatic cancer in our patient who had suffered from CP for 15 years, although our drainage procedure may reduce the risk for pancreatic cancer. When patients will need a second surgery for pancreatic cancer in the future after our triple drainage procedure, pancreaticojejunostomy or distal pancreatectomy could be performed without reconstruction (Fig. 8). Outflow pathways for bile juice, pancreatic fluid, and foods remain intact. Considering these aspects, our procedure may be a reasonable and safe option.

## Conclusion

Our drainage procedure for chronic pancreatitis showed a favorable postoperative course. In addition, from the perspective of the risk for future pancreatic cancer, our procedure may be considered a reasonable option.

## Data Availability

Data sharing is applicable to this article.

## Abbreviations

AST: Aspartate aminotransferase
ALT: Alanine aminotransferase
ALP: Alkaline phosphatase
γGTP: Gamma-glutamyl transpeptidase
T-Bill: Total bilirubin
SMV: Superior mesenteric vein
IMV: Inferior mesenteric vein
SV: Splenic vein
LRV: Left renal vein
LGV: Left gastric vein
IVC: Inferior vena cava
LGEV: Left gastroepiploic veins
PPI: Proton-pump inhibitors
HVPG: Hepatic venous pressure gradient
